# Potential Therapeutic Effects of Lipoic Acid on Memory Deficits Related to Aging and Neurodegeneration

**DOI:** 10.3389/fphar.2017.00849

**Published:** 2017-12-12

**Authors:** Patrícia Molz, Nadja Schröder

**Affiliations:** ^1^Graduate Program in Medicine and Health Sciences, Faculty of Medicine, Pontifical Catholic University, Porto Alegre, Brazil; ^2^Neurobiology and Developmental Biology Laboratory, Faculty of Biosciences, Pontifical Catholic University, Porto Alegre, Brazil

**Keywords:** memory, lipoic acid, neuroprotection, aging, neurodegenerative disorders

## Abstract

The aging process comprises a series of organic alterations, affecting multiple systems, including the nervous system. Aging has been considered the main risk factor for the advance of neurodegenerative diseases, many of which are accompanied by cognitive impairment. Aged individuals show cognitive decline, which has been associated with oxidative stress, as well as mitochondrial, and consequently energetic failure. Lipoic acid (LA), a natural compound present in food and used as a dietary supplement, has been considered a promising agent for the treatment and/or prevention of neurodegenerative disorders. In spite of a number of preclinical studies showing beneficial effects of LA in memory functioning, and pointing to its neuroprotective potential effect, to date only a few studies have examined its effects in humans. Investigations performed in animal models of memory loss associated to aging and neurodegenerative disorders have shown that LA improves memory in a variety of behavioral paradigms. Moreover, cell and molecular mechanisms underlying LA effects have also been investigated. Accordingly, LA displays antioxidant, antiapoptotic, and anti-inflammatory properties in both *in vivo* and *in vitro* studies. In addition, it has been shown that LA reverses age-associated loss of neurotransmitters and their receptors, which can underlie its effects on cognitive functions. The present review article aimed at summarizing and discussing the main studies investigating the effects of LA on cognition as well as its cell and molecular effects, in order to improve the understanding of the therapeutic potential of LA on memory loss during aging and in patients suffering from neurodegenerative disorders, supporting the development of clinical trials with LA.

## Introduction

Aging is a multifactorial process that involves genetics, lifestyle, and environmental factors (Hagen et al., 1999; Savitha and Panneerselvam, 2006; Lopez-Otin et al., 2013; Kennedy et al., 2014). During aging, biological processes promote the gradual loss of the individual's ability to maintain homeostasis, followed by a progressive deterioration in biochemical and physiological functions of the organism, increasing the susceptibility to diseases associated with aging (Arivazhagan et al., 2001a; Kumaran et al., 2005; Singh et al., 2015). Cognitive function also declines with age (Liu, 2008).

Aging has been associated to a more oxidized state in the redox balance (Jones and Sies, 2015), and the central nervous system becomes vulnerable to oxidative stress (Arivazhagan et al., 2002; Kidd, 2005; Ferreira et al., 2013; Zuo and Motherwell, 2013), defined as an unbalanced redox signaling, related to increased amounts of oxidants and ineffective antioxidant defenses (Go and Jones, 2017). In this context, nutrition can be considered a critical life-style factor that impacts the development and progression of neurodegenerative diseases (Virmani et al., 2013). Dietary supplementation with mitochondrial nutrients could promote natural neuroprotective effects, delaying the onset or progression of cognitive dysfunction and neurodegenerative diseases (Miquel, 2002; Abadi et al., 2013; Di Domenico et al., 2015; Mehrotra et al., 2015).

Over the years, lipoic acid (LA) has received increased attention as a nutritional supplement with therapeutic potential in the treatment or prevention of different pathologies (Shay et al., 2009; Rochette et al., 2013; Park et al., 2014), such as neurodegenerative diseases (Packer et al., 1997; Hager et al., 2001; Holmquist et al., 2007; Farr et al., 2012). LA has been shown to improve mitochondrial function (Kidd, 2005; Zhang et al., 2010; Zuo and Motherwell, 2013; Hiller et al., 2016), in addition to protect from cognitive dysfunction associated to aging and neurodegenerative diseases (Hager et al., 2007; Moreira et al., 2007; Liu, 2008). Thus, this study aims to review and discuss main findings showing potential memory-improving effects, as well as neuroprotective effects of LA, giving support to its use as an adjuvant in the treatment of neurodegenerative disorders. For this, we focused in discussing experimental studies addressing behavioral evaluations and cellular and molecular effects of LA.

## Lipoic acid

LA (1,2-dithiolane-3-pentonoico acid, thioctic acid) (Shay et al., 2009) was discovered in 1937 by Snell et al. (1937) and characterized by Reed et al. (1951). LA has been considered a powerful micronutrient presenting a range of pharmacological properties (Rochette et al., 2013; Koufaki, 2014; Park et al., 2014); however, many aspects of LA effects still need to be clarified, especially its application in the treatment and prevention of neurodegeneration.

### Chemistry of LA

**Figure 1:**
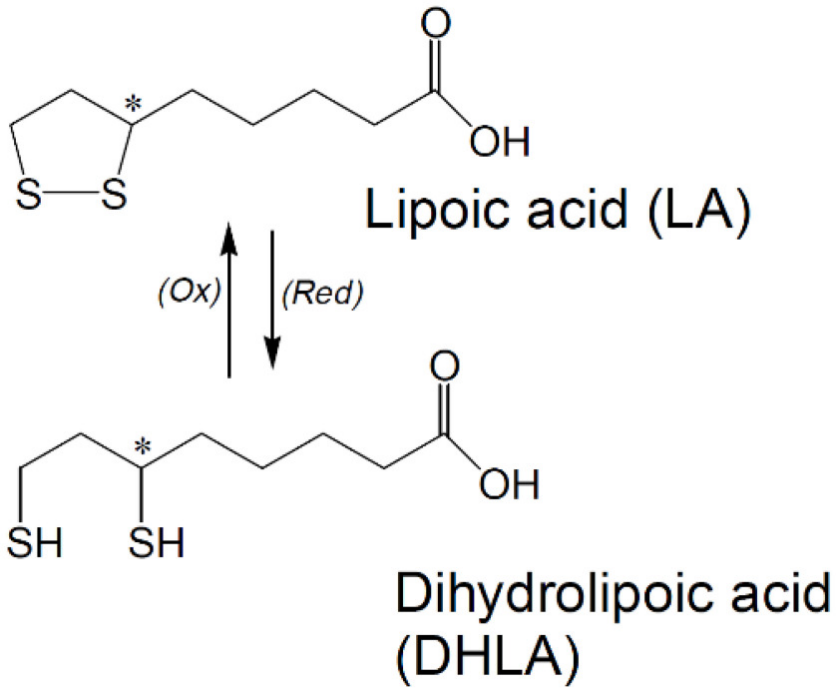
Chemical structure of lipoic acid (LA) and dihydrolipoic acid (DHLA). ^*^Indicates a chiral Carbon atom.

LA is a low molecular weight dithiol with a chiral center containing eight-carbon disulfide in its structure (Figure 1; De Araujo et al., 2011). It is naturally occurring in all prokaryotic and eukaryotic cells (Bast and Haenen, 2003; Park et al., 2014). LA acts as co-factor in multienzyme complexes in the mitochondria, such as pyruvate dehydrogenase and α-ketoglutarate dehydrogenase (Holmquist et al., 2007; Ghibu et al., 2009). A substantial part of LA is reduced to dihydrolipoic acid (DHLA) by lipoamide dehydrogenase (E3 component of the pyruvate dehydrogenase complex and α-ketoglutarate dehydrogenase) with involvement of the NADH and NADPH system (Arivazhagan et al., 2001b; Bilska et al., 2007). Reduction of LA to DHLA may also be completed by other cellular reducing systems, including NAD(P)H-driven enzymes, such as thioredoxin reductases (Rochette et al., 2013). LA also contains an asymmetric carbon resulting in two optical isomers, the S form and the R form, with the former being synthesized endogenously (De Araujo et al., 2011).

### Dietary sources, metabolism, toxicity, and nutritional recommendations

LA may be obtained from the diet (Packer et al., 1997; Hagen et al., 1999; Morikawa et al., 2001; Ghibu et al., 2009) and due to its capability of endogenous synthesis it is not considered a vitamin (Packer et al., 1995, 2001; Bast and Haenen, 2003), but is structurally considered a member of the vitamin B family (Xing et al., 2015). When ingested as a nutritional supplement preferably in the racemic mixture form, LA contains two isomers (1:1 of R-LA and S-LA), in which S-LA may prevent polymerization of R-LA and thus increases the bioavailability of the later (De Araujo et al., 2011).

LA is found in both vegetable and animal-based foods, identified as lipoyl-lysine (coupling of LA to specific lysine residues; Ghibu et al., 2009; Shay et al., 2009; Rochette et al., 2013; Li et al., 2015). R-LA is most abundantly found in vegetables such as spinach, broccolis, and tomatoes, which contain, respectively, 3.15 ± 1.11, 0.94 ± 0.25, and 0.56 ± 0.23 × 10^−3^g lipoyl-lysine (gram dry weight). In foods of animal origin, the highest amounts of lipoyl-lysine are found in bovine kidney, heart, and liver, containing, respectively, 2.64 ± 1.23, 1.51 ± 0.75, and 0.86 ± 0.33 × 10^−3^ g lipoyl-lysine (gram dry weight) (Lodge and Packer, 1999).

The biochemistry and pharmacokinetics of LA have been extensively reviewed elsewhere (Packer et al., 1995; Bustamante et al., 1998; Cremer et al., 2006b; Shay et al., 2009; Rochette et al., 2013). In summary, either from food or nutritional supplement sources, LA is rapidly absorbed, metabolized and excreted (Moini et al., 2002; Rochette et al., 2013). LA is absorbed rapidly from the gastrointestinal tract (Hagen et al., 1999; Jalali-Nadoushan and Roghani, 2013), up to 93% of a dose administered orally is absorbed in the gastrointestinal tract, (Cremer et al., 2006b) and is clearly subject to considerable pre-systemic elimination (Hagen et al., 1999). Approximately 27–34% LA orally administered is available for absorption by the tissues (Hagen et al., 1999) and the liver is one of the main clearance organs because it has a high absorption and storage capacity (Bustamante et al., 1998; Hagen et al., 1999).

Studies have shown that the gastrointestinal absorption of LA is highly variable and its efficiency appears to be reduced when ingested in the diet, suggesting that the absorption of LA competes with other nutrients (Packer et al., 2001; Shay et al., 2009; Rochette et al., 2013). Gastrointestinal uptake of LA is fast and its presence in the plasma is followed by a fast clearance (Shay et al., 2009). The plasmatic half-life of LA is 30 min. Endogenous plasma levels of LA and DHLA in humans are, respectively, 1–25 × 10^−9^ g/mL and 30–140 × 10^−9^ g/mL (Ghibu et al., 2009; Rochette et al., 2013). Urinary excretion is maximal 3–6 h following LA administration. Approximately 45% is excreted in the urine during the first 24 h and only 3% is excreted in the feces. Moreover, only a small amount of the administered LA is excreted in the unaltered form (Bustamante et al., 1998).

There are no recommendations for daily LA intake in humans. However, the safety of LA was determined in acute and sub-chronic toxicity studies as well as its mutagenicity/genotoxicity in *in vitro* and *in vivo* studies. In dogs, a LD_50_ of 400–500 mg LA/kg b.w. has been reported (Packer et al., 1995). In rats a LD_50_ of 2,000 mg/kg b.w. was described, with some rats presenting signs of reduced well-being, including sedation, apathy, piloerection, hunched posture, and/or eye closure (Cremer et al., 2006b). On the other hand, studies evaluating oral LA supplementation up to 60 mg/kg per day in rats showed no adverse effects concerning body weight, histopathological findings, and blood analyses (Cremer et al., 2006a,b). Therefore a NOAEL (no observed adverse effect level) for rats of 60 mg/kg/day for long-term LA supplementation was provided. Clinical trials using LA to assess adverse health effects in humans were performed in doses up to 2,400 mg/day with no reported adverse effects vs. placebo (Shay et al., 2009). In spite of that, the exact doses that could induce adverse human health effect are still to be set up.

## Neurodegenerative disorders

Neurodegenerative diseases are a heterogeneous group of disorders described by progressive and selective neuronal death with degeneration of specific brain regions (Arivazhagan and Panneerselvam, 2002; Lin and Beal, 2006; Savitha and Panneerselvam, 2006), often associated with abnormal deposits of proteins in neurons or extracellularly (Chen et al., 2012). Neurodegeneration is characterized by its insidious and chronic progressive onset and aging has been considered the main risk factor (Lin and Beal, 2006; Chen et al., 2012; Virmani et al., 2013; Irwin et al., 2016).

During aging, deleterious changes accumulate, causing the gradual decline of the biochemical and physiological functions (Arivazhagan and Panneerselvam, 2002; Kumaran et al., 2005; Santos et al., 2013). Moreover, aging individuals are susceptible to degeneration of selective brain regions (Aliev et al., 2009), increasing the incidence of diseases such as Alzheimer's, Parkinson's, Huntington's diseases, among others (Lin and Beal, 2006; Bagh et al., 2011; Irwin et al., 2016).

Over the last decades, a wide range of studies have shown that progression of neurodegeneration is associated with increased DNA damage (partly is attributed to an imbalance between antioxidant and prooxidant factors; Chen et al., 2012; Kim et al., 2015). In addition, mitochondrial decline, leading to cognitive dysfunction (Liu, 2008; Aliev et al., 2009; Bishop et al., 2010; Irwin et al., 2016) as well as aggregation of oxidized proteins, accumulation of metals, inflammation and excitotoxicity have been reported (Moreira et al., 2007). Oxidative stress and mitochondrial dysfunction are interrelated mechanisms that play a central role in aging brain (Santos et al., 2013), since the continuous generation of reactive oxygen species (ROS), mainly the superoxide anion (${\text{O}}_{2}^{-•}$) at complexes I and III of the mitochondrial respiratory chain, throughout life produces a sustained oxidative stress by aging, possibly resulting in cognitive impairments (Limoli et al., 2004; Kumaran et al., 2005; Bagh et al., 2011). Although the exact mechanisms underlying the effects of unbalanced redox signaling are not completely elucidated, studies suggest that it plays a role in the pathogenesis of neurodegenerative disorders (Liu et al., 2017).

Brain aging has been related to structural alterations and inflammation, accompanied by cognitive and memory dysfunctions (Bagh et al., 2011; Pizza et al., 2011; Thakurta et al., 2014). With the aging of the world population and the increasing life expectancy, the risk of developing neurodegenerative diseases is higher than ever, as they are affecting millions of people each year in epidemic proportions (Santos et al., 2013; Irwin et al., 2016). As a result, an inevitable socioeconomic burden on our health care systems will occur. Effective prophylactic and therapeutic treatments are urgent for this group of seemingly inexorable diseases. In this review we will focus on discussing the ameliorating effects of LA on cognitive deficits observed in animal models of aging and neurodegenerative disorders.

## Effects of LA in experimental models of memory deficits associated to neurodegenerative disorders

### Behavioral studies

#### Alzheimer's disease

Alzheimer's disease (AD) is the most common neurodegenerative disorder that causes dementia and affects middle to old-aged individuals (Hager et al., 2001; Gonzalez et al., 2014; Irwin et al., 2016). AD is characterized by progressive loss of cognitive functions, including memory, language, and reasoning (Di Domenico et al., 2015).

Studies have investigated the effects of LA in experimental AD models (Jesudason et al., 2005; Siedlak et al., 2009; Ahmed, 2012; Sancheti et al., 2013). For instance, Quinn et al. (2007) evaluated the chronic dietary supplementation with LA on hippocampus-dependent memory of aged Tg2576 mice, a transgenic model of cerebral amyloidosis associated with AD. LA treatment was shown to reduce hippocampal-dependent memory deficits, significantly improving learning and memory in the Morris water maze in comparison to Tg2576 mice that did not receive LA. However, no significant differences in β-amyloid levels were found between Tg2576 mice that received LA in comparison to the ones that did not receive LA, indicating that chronic LA supplementation in the diet can ameliorate hippocampal memory impairments in Tg2576 mice without any effect on β-amyloid levels or plaque deposition.

Another study assessed the effects of LA in senescence-accelerated mouse prone 8 (SAMP8) mice, associated to learning and memory impairments, and showed that LA can improve memory, in different paradigms (Farr et al., 2012). In object recognition, results indicated that mice that received LA presented a higher memory index than vehicle-treated mice. When memory was tested in the Barnes maze, results indicated that LA-treated mice spent more time by the target, and made fewer errors than controls, but did not present differences in speed in traversing the maze (distance/time) during training.

On the other hand, Siedlak et al. (2009) investigated young and aged mice overexpressing amyloid-β precursor protein (APP) and controls and showed that administration of R-LA had little effect on Y-maze performance. The authors concluded that, although oxidative stress has been proposed to mediate amyloid pathology and cognitive decline in aging, long-term LA administered within tolerable nutritional levels, presented limited benefit.

#### Parkinson's disease

Parkinson's disease (PD) is the second most frequent neurodegenerative disorder in aging individuals and features motor symptoms related to dopaminergic neuronal loss in the *substantia nigra*, which results in decreased striatal dopaminergic terminals (Beal, 2003; De Araujo et al., 2011). Studies have shown that, in addition to rescuing cognitive deficits, LA is also able to ameliorate motor impairment related to PD. The effects of LA were examined in a rat model of PD induced by rotenone. The effect of LA (50 mg/kg/day, p.o.) was evaluated after the administration of rotenone in the open-field and square bridge tests. The authors reported that LA improved rotenone-induced behavioral deficits. In the open-field test, LA significantly increased the ambulation frequency, increased the number of stops, elevated the activity index and lessened the inactive sittings, but did not increase the rearing frequency in comparison to the group that received rotenone. In the Square bridge test, treatment with LA protected the rats from falling as compared to rotenone group (Zaitone et al., 2012).

Jalali-Nadoushan and Roghani (2013) investigated the effect of LA (at doses of 50 and 100 mg/kg) in a 6-hydroxydopamine (6-OHDA)-induced model of hemi-parkinsonism and observed significantly attenuated rotations on behavioral testing, induced by both doses.

The effects of LA in lipolysaccharide (LPS)-induced inflammatory PD model were also evaluated (Li et al., 2015), and the results showed that LA treatment (100 mg/kg/d) partially improved motor dysfunction. No significant recovery was observed in dyskinesia in PD mice that received LA. However, a significant amelioration was observed in the adhesive removal test, in which LA treatment significantly decreased the reaction time in comparison to the LPS group.

#### Huntington's disease

Huntington's disease (HD) is a chronic neurodegenerative disease and a hereditary autosomal-dominant disorder of the central nervous system caused by a single genetic mutation (Ross et al., 2014), characterized by neuronal death in caudate and putamen and in the cerebral cortex, and to a lesser extent in hippocampus and subthalamic nucleus (Mehrotra et al., 2015). This disorder is classically characterized by motor symptoms and cognitive and behavioral features (Ross et al., 2014). A HD model that has been easily replicated in animals is based on the treatment with 3-nitropropionic acid (3-NP), which promotes development of mitochondrial dysfunctions leading to bioenergetic failure (energy impairment, oxidative stress, and excitotoxicity). A study by Mehrotra et al. (2015) investigated the effects of LA in 3-NP-induced HD in rats. Administration of LA improved spatial memory acquisition and retrieval assessed using the Morris water maze. Analysis of time taken and distance traveled to find the platform in the target quadrant revealed that LA supplementation for 21 days to 3-NP–treated animals resulted in a lower latency and the distance traveled was also reduced. In addition, the average number of platform crossings in the probe trial was increased in 3-NP treated animals that received LA. Thus, the authors demonstrated that LA supplementation improved spatial memory by ameliorating the iron- and copper-induced oxidative injury observed in age-related disorders. In the Y-maze test, animals display a preference to explore the novel arm of the maze, making fewer entrances in the previously explored arm, due to spontaneous alternation. The authors showed that 3-NP–treated animals traveled a significantly lower distance, assessed by the number of entries in the novel arm, and that supplementation with LA reversed these deficits.

#### Aging models

Consistent evidence indicate that memory is affected by aging in rodents as well as in humans. A study by Liu et al. (2002a) investigated the effects of LA supplementation (0.1% in the diet) on spatial memory tested in the Morris water maze, and temporal memory using the peak procedure (time-discrimination procedure) in old rats. Results showed that LA supplementation alone or combined with another mitochondrial metabolite, acetyl-l-carnitine, improved both spatial and temporal memory. In 12-month old SAMP-8 mice, chronic LA administration improved cognition in both the T-maze footshock avoidance paradigm and the lever press appetitive task without inducing non-specific locomotor effects (Farr et al., 2003).

LA has also been reported to improve behavior of aged mice in an open-field memory test (Stoll et al., 1993, 1994), in a Morris water maze test (Stoll et al., 1994; Liu et al., 2002a). LA also and ameliorated acquisition and retrieval in a dose-dependent manner, in old female NMRI mice, in the active avoidance learning test (Stoll et al., 1994).

#### Other models of neurotoxicity

Cui et al. (2006) evaluating a concomitant treatment with LA and d-Galactose exposure (used to induce memory loss and neurodegeneration) verified that LA ameliorated memory dysfunction in the Morris water maze task. Another study reported the neuroprotective effects of LA in neurotoxicity model induced by AlCl_3_ administration to mice (Mahboob et al., 2016). LA enhanced fear memory and social novelty preference in comparison to the AlCl_3_-treated group.

In summary, current evidence indicates that LA is able to improve memory, reversing impairments associated to a variety of experimental models of neurodegenerative disorders, and exposure to neurotoxicants, as well as normal aging. Table 1 summarizes *in vivo* studies investigating the neuroprotective effects of LA on behavioral parameters. Furthermore, LA was also shown to act as a memory-improving molecule in different learning and memory paradigms, including aversive, spatial, and recognition memory.

**Table 1:** Summary of studies testing the effects of LA on behavioral parameters in animal models.

**Experimental model**	**Regimen of treatment**	**Effects**	**References**
Aging	Chronic, α-LA (100 mg/kg body weight, 15 days, orally	Improved spatial memory in aged animals	(Stoll et al., 1993, 1994)
Aging	Chronic, fed an AIN-93M diet with R-LA (0.5% w/w) for 2 wk.	Improved ambulatory activity in old and young animals.	(Hagen et al., 1999)
Aging	R-α-LA [0.2% or 0.1% (wt/wt) in diet] for 7 weeks	Improved spatial and temporal memory in old animals	(Liu et al., 2002a)
SAMP8 accelerated aging mouse	Chronic, LA (100 mg/kg), subcutaneously for 4 weeks	Improved memory in the T-maze footshock avoidance paradigm and the lever press appetitive task in old animals	(Farr et al., 2003)
Aging model	Chronic, α-LA (100 mg/kg body weight) daily, 7 weeks, intraperitoneally	Improved spatial memory	(Cui et al., 2006)
Radiation-induced cognitive dysfunction	Sub-chronic, LA (200 mg/kg bw) intraperitoneally for 5 days	Improved spatial memory	(Manda et al., 2007)
Model of cerebral amyloidosis	Chronic, fed an α-LA-containing diet (0.1%) for 6 months	Improved learning and memory retention; exhibited more context freezing	(Quinn et al., 2007)
Model of amyloid-β protein precursor-over-expressing	Chronic, LA (diet 30 mg/kg per day) for 10 months	No effects on spatial memory	(Siedlak et al., 2009)
Model of AD using SAMP8 mice	Chronic, LA (100 mg/kg), subcutaneously for 4 weeks	Improved spatial memory in old animals	(Farr et al., 2012)
Model of rotenone-induced parkinsonism	Sub-chronic, α-LA (50 mg/kg/day/12 doses[12 days], po)	Improved spatial memory	(Zaitone et al., 2012)
6-OHDA model of hemi-parkinsonism in rats	50 and/or 100 mg/kg 24 h before of surgery	Attenuated rotational behavior	(Jalali-Nadoushan and Roghani, 2013)
Model of LPS-induced Parkinson's disease	Sub-chronic, LA (100 mg/kg/d) administered ip for 30 days	Improved motor dysfunction	(Li et al., 2015)
Model 3-NP–induced HD	Chronic, α-LA (50 mg/kg), intraperitoneally for 21 days	Improved motor dysfunction	(Mehrotra et al., 2015)
AlCl_3_-induced neurotoxicity	Sub-chronic, α-LA mixed in diet (200 ppm = dose of 25 mg/kg/day) for 12 days	Enhanced fear memory and social novelty preference	(Mahboob et al., 2016)

*3-NP, 3-nitropropionic acid; AD, Alzheimer's disease; AlCl3, Aluminum chloride; HD, Huntington's disease; LPS, lipolysaccharide; 6-OHDA, 6-hydroxydopamine*.

LA has been tested in humans, in studies by Hager and coworkers (Hager et al., 2001, 2007), as a treatment option for AD. The authors examined the effect of LA for 24 and 48 months and observed that the treatment lead to a stabilization of cognitive function, verified by unchangeable records in two neuropsychological tests, mini-mental state examination (MMSE) and the AD assessment score, cognitive subscale (ADAScog).

## Putative mechanisms underlying LA-induced neuroprotective effects

*In vivo* as well as *in vitro* studies have been performed in order to characterize cellular and molecular effects of LA underlying its memory-ameliorating activities (Table 2). The effects of LA on oxidative markers in various brain regions have been discussed in different studies in animals models of aging and neurodegenerative diseases (Cui et al., 2006; Ferreira et al., 2009; Militao et al., 2010; Farr et al., 2012). LA administration decreases lipid peroxidation evaluated by MDA (Arivazhagan and Panneerselvam, 2000; Arivazhagan et al., 2002; Liu et al., 2002b; Ferreira et al., 2009; Militao et al., 2010; Farr et al., 2012) in different brain regions, and elevates the activities of antioxidants such as ascorbate (vitamin C), α-tocoferol (vitamin E) (Arivazhagan and Panneerselvam, 2000), glutathione (GSH) (Arivazhagan and Panneerselvam, 2000; Farr et al., 2012), superoxide dismutase (SOD) activity (Arivazhagan et al., 2002; Cui et al., 2006; Militao et al., 2010), catalase (CAT) (Arivazhagan et al., 2002; Militao et al., 2010), glutathione peroxidase (GSH-Px) (Arivazhagan et al., 2002; Militao et al., 2010), glutathione redutase (GR) (Arivazhagan et al., 2002), glucose-6-P-dehydrogenase (G6PDH) (Arivazhagan et al., 2002). Moreover, administration of LA reversed the augmentation of protein carbonyls levels in a radiation-induced cognitive dysfunction model (Manda et al., 2007), and decreased the protein carbonyls levels in aged SAMP8 mice (Farr et al., 2003).

**Table 2:** Summary of *in vivo* studies testing cellular and molecular effects of LA.

**Experimental model**	**Regimen of treatment**	**Effects**	**References**
Aging	Chronic, α-LA (100 mg/kg body weight, orally for 15 days	LA alleviated age-related NMDA receptor deficits (B_max_)	Stoll et al., 1993
Aging	Acute and Chronic, DL-α-LA (100 mg/kg body weight/day), 7 or 14 days, young and aged rats, intraperitoneally	↓ lipid peroxidation, ↑ levels of antioxidants in various brain regions	Arivazhagan and Panneerselvam, 2000
Aging	α-LA (300 mg/kg/day) for 6weeks	↓ mtDNA deletions associated with aging	Seidman et al., 2000
Aging	Acute and Chronic, DL-α-LA (100 mg/kg body weight/day), 7 or 14 days, young and aged rats, intraperitoneally	↑levels of neurotransmitters (dopamine, serotonin and norepinephrine) in various brain regions	Arivazhagan and Panneerselvam, 2002
Aging	Acute and Chronic, DL-α-LA (100 mg/kg body weight/day), 7 or 14 days, young and aged rats, intraperitoneally	↓ level of lipid peroxidation, ↑activities of antioxidant enzymes in various brain regions	Arivazhagan et al., 2002
Aging	R-α-LA [0.2% or 0.1% (wt/wt) in diet] for 7 weeks	↓ reduced the extent of oxidized RNA, reversed age-associated mitochondrial structural decay	Liu et al., 2002a
Aging	R-α-LA [0.2% (wt/wt) in diet] for 7 weeks	Inhibited lipid peroxidation but did not decrease iron and copper levels	Liu et al., 2002b
SAMP8 accelerated aging mice	Chronic, LA (100 mg/kg), subcutaneously for 4 weeks	↓Protein carbonyl levels, ↑W/S ratio, ↓TBARS levels	Farr et al., 2003
Abeta peptide vaccination-induced inflammation	Acute, LA (100 mg kg −1 body weight), intraperitoneally for 4 days	↑ levels of 5-HT, DA and NE and the concentration of 5-HIAA and HVA gradually returned to normal	Jesudason et al., 2005
SAMP8 accelerated aging mice	Chronic, LA as a racemic mixture (100 mg/kg body weight) daily, 4 weeks, subcutaneously	↑ brain proteins (neurofilament triplet L protein, α-enolase, and ubiquitous mitochondrial creatine kinase), ↓ specific carbonyl levels of the three brain proteins (lactate dehydrogenase B, dihydropyrimidinase-like protein 2, and α-enolase)	Poon et al., 2005
Aging	Chronic, LA (0.2% [w/w]) for 2 weeks	↓cerebral iron levels, antioxidant status and thiol redox state improved markedly	Suh et al., 2005
Adult male C57BL/6 mice with D-gal administration	Chronic, α-LA (100 mg/kg body weight) daily, 7 weeks, intraperitoneally	Ameliorated neurodegeneration in the hippocampus, ↓ peripheral oxidative damage, ↑ T-AOC and T-SOD, no effect on GSH-Px, ↓ caspase-mediated apoptosis, ↑ neurogenesis and neuron migration, ↓oxidative	Cui et al., 2006
Reserpine rat model of PD	Acute, LA (50 mg/kg) administered twice, 30 min before and after reserpine injection, intraperitoneally	↑ concentration of GSH and ↓GSSG level in the striatum, ↑ GSH level and no changes in GSSG content in the prefrontal cortex, ↓ NO concentrations, ↑ Enzymatic activities of GPx and GST in the striatum	Bilska et al., 2007
MPTP model of PD	Acute, α-LA (22 mg/kg body weight, sc) twice daily, concomitant with MPTP	Abolished the activation of ASK1 and phosphorylation of downstream kinases, MKK4, and JNK and prevented the down-regulation of DJ-1 and translocation of Daxx to the cytosol; attenuated dopaminergic cell loss in SNpc	Karunakaran et al., 2007
Cognitive impairment induced by radiation	Sub-chronic, LA (200 mg/kg bw) intraperitoneally for 5 days	Protected against augmentation of protein carbonyls and TBARS in cerebellum; intact cytoarchitecture of cerebellum, higher counts of intact Purkinje cells and granular cells; T-SH, NP-SH, PB-SH contents of cerebellum and plasma FRAP was inhibited	Manda et al., 2007
Aging	Chronic, α-LA (100 mg/kg body weight/day dissolved in alkaline saline) for 30 days	Old rats: ↓ levels of mitochondrial LPO, 8-oxo-dG and oxidized glutathione and enhanced reduced glutathione, ATP, lipoic acid and ETC complex activities; Young rats: ↓ levels of LPO, 8-oxo-dG and oxidized glutathione and ↑levels of reduced glutathione, ATP, lipoic acid, TCA cycle enzymes and ETC complex activities.	Palaniappan and Dai, 2007
Tg2576 AD mouse model	Chronic, fed an α-LA-containing (0.1%) for 6 months	No effects on β-amyloid levels or plaque deposition	Quinn et al., 2007
Aging	Acute, LA (10, 20 or 30 mg/kg, i.p.), 24 h	↓ lipid peroxidation level, no alteration was observed in SOD activity, ↑ DA and NE, ↓ 5-HT and their metabolites 5-HIAA, the metabolites (DOPAC and HVA) did not differ in hippocampus	Ferreira et al., 2009
Tg2576 AD mouse model	Chronic, LA (diet 30 mg/kg per day) for 10 months	↓expression of HO-1 and protein-bound HNE, ↓ HO-1 around amyloid plaques, ↓ protein-bound HNE expression surrounding Aβ plaques, Redox active iron accumulation was specifically co-localized with Aβ plaques in the hippocampus and cortical regions	Siedlak et al., 2009
Pilocarpine-induced seizures	Acute, LA (20 mg/kg, ip) for 30 min	↓ lipid peroxidation and nitrite concentrations and ↑SOD, CAT and GPx activities in striatum	Militao et al., 2010
AlCl_3_ rat model of AD	Chronic, α-LA (100 mg/kg/day for 3 months) after AlCl_3_ (100 mg/kg b.wt/day for 4 months), orally.	↓ AChE activity, ↓ inflammation, ↑ neuronal and regeneration features	Ahmed, 2012
SAMP8 accelerated aging mice	Chronic, LA (100 mg/kg), subcutaneously for 4 weeks	↑GSH, ↓MDA, ↓GPx	Farr et al., 2012
Rotenone rat model of PD	Sub-chronic, α-LA (50 mg/kg/day/12 doses[12 days], po)	↑striatal dopamine level, no effect on striatal ATP level, ↓ level of lipid peroxides and ↓protein carbonyls in rat brains, ↑tissue GSH, improved injury to mtDNA and normalization of the mtDNA content, ↑ percentage of SNpc dopaminergic neurons, ↑ number of Nissl stained neurons	Zaitone et al., 2012
DBA/2J mouse model of glaucoma	60 mg/kg body weight (bw)/day for the intervention study and 100 mg/kg bw/day for the prevention study in diet for 24 months	↑antioxidant gene and protein expression, ↑protection of RGCs and improved retrograde transport, ↓ lipid peroxidation, ↓ protein nitrosylation, ↓ DNA oxidation in the prevention and intervention paradigms.	Inman et al., 2013
Unilateral intrastriatal 6-OHDA-lesioned rats	50 and/or 100 mg/kg 24 h before surgery	Prevented loss of SNC neurons, ↓ levels of MDA and nitrite	Jalali-Nadoushan and Roghani, 2013
3 × Tg-AD	Chronic, LA (0.23% w/v in drinking water) for 4 weeks	↑brain glucose uptake; ↑ in the total GLUT3 and GLUT4 in the old mice; activation of the insulin receptor substrate and of the PI3K/Akt signaling pathway; changes in synaptic function (↑I/O) and LTP.	Sancheti et al., 2013
Arsenic-dichlorvos exposed rats	Chronic, α-LA (50 mg/kg/day for 10 months), orally.	Oxidative stress and cholinergic dysfunction was protected	Dwivedi et al., 2014
Lipolysaccharide (LPS)-induced inflammatory PD model	Sub-chronic, LA (100 mg/kg/d) administered ip for 30 days	Protected dopaminergic neurons loss, ↓α-synuclein accumulation in the substantia nigra, inhibited the activation of NF-κB and expression of pro-inflammatory molecules in M1 microglia	Li et al., 2015
3-NP-induced HD model of	Chronic, α-LA (50 mg/kg), intraperitoneally for 21 days	Restored respiratory chain enzyme activities, CAT activity was improved, normalized of mitochondrial appearance, stimulated the repair of mitochondrial membranes and restored functionality to impaired mitochondria, ↓lipid peroxidation, ↓protein carbonyls, ↓ROS and nitrite levels, ↓cytosolic levels of cytochrome c, ↓activities of caspase-3 and 9.	Mehrotra et al., 2015
AlCl_3_-induced neurotoxicity mouse model	Sub-chronic, α-LA mixed in diet (200 ppm = dose of 25 mg/kg/day) for 12 days	Improves the expression of muscarinic receptors (M1 and M2) and choline acetyltransferase	Mahboob et al., 2016

*3-NP, 3-nitropropionic acid; 5-HIAA, 5-hydroxyindoleacetic acid; 5-HT, 5-hydroxytryptamine; 5-HT, serotonina; 6-OHDA, 6-hydroxydopamine; 8-oxo-dG, monoclonal anti-8-hydroxyguanine; AChE, acetylcholinesterase; AD, Alzheimer's disease; AlCl_3_, Aluminum chloride: ASK1, apoptosis signal regulating kinase 1; Aβ, amyloid-β fibrils; CAT, catalase activity; DA, dopamine; D-gal, D-galactose; DOPAC, 3,4-hydroxyphenylacetic acid; ETC, electron transport chain; FRAP, ferric reducing power; GLUT3, glucose transporter 3; GLUT4, glucose transporter 4; GPx, glutathione peroxidase; GSH, reduced glutathione; GSH-Px, glutathione peroxidase; GSSG, glutathione disulfide; GST, glutathione-S-transferase; HNE, 4-hydroxynonenal; HO-1, heme oxygenase-1; HVA, homovanillic acid; I/O, input/output; JNK, Jun N-terminal kinase; LPO, lipid peroxidation; LPS, Lipolysaccharide; LTP, long term potentiation; M1,Type 1 macrophages/microglia; M2, Type 2 macrophages/microglia; MDA, malondialdehyde; MKK4, mitogen-activated protein kinase kinase 4; MPTP, 1-methyl-4-phenyl-1, 2, 3, 6-tetrahydropyridine; mtDNA, mitochondrial DNA; NE, norepinephrine; NF-κB, nuclear factor-κB; NMDA receptor, N-methyl-D-aspartate receptor; NO, nitric oxide; PD, Parkinson's disease; RGC, retinal ganglion cell; ROS, reactive oxygen species; SNC, substantia nigra pars compacta; SNpc, substantia nigra pars compacta; SOD, total superoxide dismutase; T-AOC, total antioxidative capabilities; TBARS, thiobarbituric acid reactive substance; T-SOD, total superoxide dismutase; W/S, weakly immobilized/strongly immobilized*.

A study by Zaitone et al. (2012) showed that LA increased striatal dopamine levels and significantly increased GSH and CAT activity in the striatum in a PD experimental model. In reserpine-treated rats, LA enhanced the amount of GSH, while diminishing GSSG levels in the striatum. Moreover, LA decreased NO concentrations in striatum and pre-frontal cortex, without significantly affecting S-nitrosothiol levels. LA also increased enzymatic activities of GPx and GST in the striatum (Bilska et al., 2007). Reserpine significantly decreased enzymatic activity of L-γ-glutamyl transpeptidase (γ-GT), while pretreatment with LA was able to restore it.

The effects of LA on oxidative stress in rotenone parkinsonian rat brains were investigated, showing that LA can reduce lipid peroxidation and protein carbonylation (Zaitone et al., 2012). LA also lowered the levels of MDA and nitrite in the 6-OHDA-induced rat model of hemi-parkinsonism (Jalali-Nadoushan and Roghani, 2013). Karunakaran et al. (2007) analyzed the protective effect of LA in the MPTP mouse model of PD, demonstrating that coadministration with LA prevents the activation of apoptosis signal regulating kinase (ASK1) signaling cascade and translocation of Daxx (death associated protein) in ventral midbrain and striatum, attenuating dopaminergic cell loss. R-LA induced significant reductions in markers of oxidative modifications in transgenic AD mice model, significantly decreasing HO-1 and protein-bound HNE levels (Siedlak et al., 2009). Inman et al. (2013) analyzed the effect of LA in the DBA/2J mouse model of glaucoma. The results showed that after 4 and 11 months of dietary LA, respectively, LA treatment increased antioxidant genes and protein expression, protected retinal ganglion cell (RGC), and improved retrograde transport. Dietary therapy also reduced lipid peroxidation, protein nitrosylation, and DNA oxidation in a retina model of glaucoma.

Accumulation of metal ions also has been associated with increased oxidative stress related with aging and neurodegenerative disorders. Suh et al. (2005) showed that LA supplementation can modulate age-related cortical iron accumulation, acting as metal chelator, thereby ameliorating age-associated oxidative stress. However, Liu et al. (2002b) showed that high concentrations of iron and copper found in old rats were not significantly decreased with LA supplementation.

There are multiple cell death mechanisms implicated in neurodegeneration. Apoptosis is a highly controlled cellular process that can be activated by two pathways: extrinsic, which is a receptor-mediated pathway, and intrinsic, which is mediated by signals from the mitochondria. Both pathways culminate at cleavage-dependent activation of aspartate-specific effector caspases (caspases-3, 6, and 7). Cui et al. (2006), using chronic systemic exposure of d-galactose in an aging model observed that a treatment with LA decreased caspase-3 protein levels and neuronal apoptosis, ameliorating neurodegeneration in the hippocampus. Manda et al. (2007), demonstrated that LA pretreatment protected against radiation. They observed that pre-treatment with LA prevented radiation-induced decreases of total, nonprotein and protein-bound sulfhydryl (T-SH, NP-SH, and PB-SH) levels in the cerebellum. Moreover, LA treatment also improved the cytoarchitecture of cerebellum, increasing the number of intact Purkinje cells and granular cells when compared to untreated irradiated mice.

Mehrotra et al. (2015) evaluated the effects of LA on mitochondrial dysfunctions in the 3-NP induced model of HD. The results showed that LA decreased malondialdehyde, protein carbonyls, reactive oxygen species and nitrite levels, and increased Mn-superoxide dismutase and CAT activity. They also found that LA improved histological and biochemical alterations, such as decreased cytosolic cytochrome c levels, caspase-3 and−9 activity and expression of apoptotic proteins (AIF, Bim, Bad, and Bax), suggesting its therapeutic efficacy in HD. LA improved activity of enzymes from the mitochondrial respiratory chain, altered cytochrome levels, increased histochemical staining of complex-II and IV, increased in-gel activity of complex-I to V, and increased mRNA expression of respiratory chain complexes.

Stoll et al. (1993) investigated the effect of LA on NMDA Receptor deficits in old female NMRI mice. The results showed that LA improved age-related NMDA receptor deficits (B_max_). No changes were observed regarding muscarinic, benzodiazepine, and α_2_-adrenergic receptor deficiencies. Thus, the authors concluded that LA-induced memory improving effects may be related to partial reparation of NMDA receptor deficits that accompany aging.

A loss of dopaminergic neurons is particularly relevant to PD, in which genetic and environmental factors are involved (Di Domenico et al., 2015; Li et al., 2015). Jalali-Nadoushana and Roghania using a rat model of hemi-parkinsonism (6-OHDA) found that LA prevented neuronal loss on the left side of the *substantia nigra pars compacta* (SNpc) (Jalali-Nadoushan and Roghani, 2013). In a study using the LPS-induced inflammatory PD model, Li et al. (2015) demonstrated that LA administration protected against dopaminergic neuron loss. Zaitone et al. (2012) observed that LA induced an increase in the number of neurons in the SNpc in rotenone parkinsonian rats. Li et al. (2015) reported that in addition to protecting against dopaminergic neuron loss, LA also decreased α-synuclein deposits in the substantia nigra (SN). Moreover, the authors showed that LA inhibited the stimulation of nuclear factor-κB (NF-κB) and expression of pro-inflammatory molecules in M1 microglia. Zaitone et al. (2012) also investigated the effect of LA on mitochondrial DNA (mtDNA) integrity and quantity in the rotenone model of PD. The results showed that LA significantly decreased rotenone-induced mtDNA damage.

Liu et al. (2002a), examined the effects of LA on mitochondrial structure, and neurodegeneration in the hippocampus, and oxidative damage to nucleic acids in the hippocampus and cortex of aged rats. Dietary administration of LA significantly reduced oxidized RNA levels and reversed mitochondrial structural deterioration induced by aging in the hippocampus. Dwivedi et al. (2014) investigated the protective efficacy of LA against co-exposure to arsenic-dichlorvos in rats. The results indicated that arsenic and dichlorvos induced oxidative stress and cholinergic dysfunction in brain, which was significantly protected by the supplementation with LA.

Seidman et al. (2000) indicated that age-associated mitochondrial impairment may be hampered by LA administration. Their results showed that mtDNA deletions associated with aging were reduced by LA and this effect appeared to be related to the mitochondrial capacity to protect and repair mtDNA against age-induced injury. Palaniappan and Dai (2007) investigated the effect of LA administration to aged rats and verified a reduction of mitochondrial lipid peroxidation, 8-oxo-dG and oxidized glutathione (GSSG) and increased GSH, ATP, and electron transport chain (ETC) complex activities in the brain.

The SAMP8 mouse strain is an experimental model that displays increased oxidative stress accompanied by memory decline associated to a rapid aging process. Proteomic analyses were used to examine differential protein expression and/or protein oxidative changes in brain samples from aged SAMP8 mice. In order to determine the mechanisms underlying LA-induced reversion of memory deficits exhibited by SAMP8 mice, Poon et al. (2005) analyzed the expression and specific carbonylation of proteins in brains from 12-month-old SAMP8 mice that received LA or vehicle. The levels of three proteins (neurofilament triplet L protein, a-enolase, and ubiquitous mitochondrial creatine kinase) were significantly increased, while protein carbonylation was reduced in lactate dehydrogenase B, dihydropyrimidinase-like protein 2, and a-enolase in aged SAMP8 mice that received LA, suggesting that, in addition to improving learning and memory, LA also can restore specific proteins in aged SAMP8 mouse brain.

Evidence indicates that deregulation in neurotransmitter systems, including decreased levels of neurotransmitters, decline in the number of receptors, and lower responsiveness to neurotransmitters can be key features of neurological disorders (Payton et al., 2005; Fidalgo et al., 2013). Arivazhagan and Panneerselvam (2002) investigated the effect of LA on levels of neurotransmitters (dopamine, serotonin, and norepinephrine), and showed that LA treatment can improve neurotransmitter function in models of neurodegenerative diseases. Jesudason et al. (2005) investigated the effect LA on levels of neurotransmitters in a model of AD by Aβ amyloid vaccination. The results showed that AD mice treated with LA exhibited enhanced levels of serotonin, dopamine, and norepinephrine, and the concentration of metabolites 5-hydroxyindole acetic acid (5-HIAA) and homovanillic acid (HVA) gradually returned to normal.

Ahmed (2012) explored the effect of LA on brain acetylcholinesterase (AChE) activity. The authors demonstrated that LA can ameliorate neurological injury related to Aβ and Al excess, by significantly restoring AChE activity. In addition, the authors showed that the treatment with LA restored the parameters of total homocysteine (tHcy), insulin, insulin like growth factor-1 (IGF-1), interlukin-1β (IL-1β) and tumor necrosis factor-α (TNF-α). Mahboob et al. (2016), analyzed the effects of LA in AlCl_3_- model of neurodegeneration, demonstrating its capacity in ameliorating cognitive functions and enhancing cholinergic system functions. LA treatment increased the expression of muscarinic receptor genes M1, M2 and choline acetyltransferase (ChaT) relative to AlCl_3_-treated group.

There are many studies examining the neuroprotective actions of LA using *in vitro* models of neurodegeneration (Tirosh et al., 1999; Li et al., 2013; Xing et al., 2015), most of which focus on AD. For example, Ono et al. (2006) investigated the effects of LA and its reduced form, DHLA, on the formation, extension, and destabilization of β-amyloid fibrils (fAβ). The results showed that both LA and DHLA inhibited fAβ formation from amyloid β, as well as their expansion, and undermined preformed fAβs in a dose dependent manner. Lovell et al. (2003) also studied the effects of LA and DHLA in neuronal cultures challenged with amyloid β-peptide (Aβ 25-35), and observed that DHLA, but not LA, significantly protected against neurotoxicity induced by amyloid β-peptide and iron/hydrogen peroxide (Fe/H_2_O_2_).

In β-amyloid-intoxicated C6 glioma cells, LA increased cell viability and MnSOD expression. The increased GSSH and decreased GSH mitochondrial levels induced by Aβ were reversed by treatment with LA (Xing et al., 2015). In addition, LA protected cortical neurons against Aβ peptide- and hydrogen peroxide-induced damage, suggesting that the neuroprotective effects were partly related to PKB/Akt signaling pathway stimulation (Zhang et al., 2001).

The study by Deuther-Conrad et al. (2001) showed that the advanced glycation end products (AGE)-induced increases in oxidized glutathione were inhibited by R-LA in SH-SY5Y human neuroblastoma cells, indicating that AGE-mediated depletion of reduced glutathione follows the production of superoxide and hydrogen peroxide. de Arriba et al. (2003) investigated the effect of R-LA, in the same types of cells, on AGE accumulation, and found that AGE-induced metabolic changes were diminished by R-LA. Tirosh et al. (1999) showed that LA protected HT4 neuronal cells against glutamate-induced cytotoxicity, by inhibiting intracellular GSH depletion, and canceled the buildup of intracellular peroxide levels following the glutamate exposure.

Kamarudin et al. (2014) showed that R-LA ameliorated glutathione over glutathione disulfide ratio, decreased intracellular ROS levels and increased mitochondrial membrane potential in NG108-15 cells. In addition, R-LA stimulated the production of an anti-inflammatory cytokine, IL-10, inactivating glycogen synthase kinase-3b (GSK-3β) and decreasing both Bax/Bcl2 and Bax/Bcl-xL ratios. Suppression of NF-κβ p65 translocation and production of proinflammatory cytokines (IL-6 and TNF-α) followed inhibition of cleaved caspase-3. Yamada et al. (2011) investigated the effects of different isomers of LAs (racemate, R-LA, and S-LA) in human neuroblastoma SH-SY5Y cells. They showed that all types of LAs were effective in preventing cell death. R-LA and S-LA also enhanced expression of genes related to anti-oxidative response such as heme oxygenase-1 (HO-1) and phase II detoxification enzymes such as NAD(P)H:Quinone Oxidoreductase 1 (NQO1).

Other studies evaluated the effect of LA on *in vitro* model of PD. Li et al. (2013) showed that pretreatment with LA significantly prevented against apoptosis of PC12 cells elicited by MPP+, and inhibited intercellular ROS levels and mitochondrial transmembrane permeability, thereby protecting dopaminergic neuronal cells against oxidative damage. Moreover, Zhang et al. (2010) demonstrated that R-LA hindered rotenone-induced mitochondrial dysfunction, oxidative damage, and α-synuclein and ubiquitin deposition in SK-N-MC human neuroblastoma cells.

### Involvement of LA in the regulation of cellular signaling pathways

LA has been proposed to exert a modulatory control on the cellular redox status. Due to its ability to be interconverted in one of its two forms—i.e., thiol, the reduced form and disulfide, the oxidized form—LA can regulate cellular redox environment by interacting with redox couples such as glutathione/glutathione disulfide, cysteine/cystine, and thioredoxin (Packer and Cadenas, 2011). LA has been described to regenerate other antioxidants, such as vitamin C and E, to increase GSH levels, and to provide modulation of proteins and transcription factors (Packer et al., 1995). Extracellular redox state is also regulated by LA, once its reduced form, DHLA, can interact with cystine, reducing it to cysteine, thereby stimulating its uptake by the cell, which in turn stimulates GSH synthesis (Han et al., 1997). Owing to the role played by LA in the regulation of thiol/disulfide redox couples, LA can be viewed as regulator of cell signaling and gene expression.

PI3K/Akt signaling pathway, critical to the regulation of cell growth, proliferation, differentiation, survival, and metabolism, has been shown to be modulated by LA. For instance, Jiang et al. (2013) have demonstrated that age-associated imbalance of PI3K/Akt was restored by LA treatment for 3 weeks in the drinking water in rats. LA treatment significantly increased Akt phosphorylation and lead to a recovery in the ratio pJNK/pAkt in cortex of aged rats. Prevention of sevoflurane-induced apoptosis by LA was accomplished through recovery of Akt and GSK3-β phosphorylation levels in the hippocampus (Ma et al., 2016). By activating PI3K/Akt signaling pathway, LA, administered for 3 days, was able to ameliorate cerebral ischemia and reperfusion-induced damage in adult rats (Dong et al., 2015). Sancheti et al. (2013) showed that LA increases brain glucose uptake and activates the insulin receptor substrate and the PI3K/Akt signaling pathway in a triple transgenic mouse model of AD (3xTg-AD), reversing the impaired synaptic plasticity and increasing input/output (I/O) and long-term potentiation (LTP).

## Concluding remarks

Compelling evidence indicates that LA displays memory-ameliorating properties in a variety of experimental models of neurodegenerative diseases, as well as in memory decline associated with aging in rodents. Studies aiming to assess the neuroprotective effects of LA on behavioral outcomes showed that LA can reduce memory deficits in different behavioral paradigms on AD (Quinn et al., 2007; Farr et al., 2012), HD (Mehrotra et al., 2015), oxidative stress (Stoll et al., 1993, 1994; Liu et al., 2002a; Farr et al., 2003; Manda et al., 2007), and age-associated cognitive dysfunction (Cui et al., 2006; Mahboob et al., 2016) models. In humans, two studies in AD patients have supported the positive cognitive effects of LA (Hager et al., 2001, 2007).

Many studies reported beneficial effects of LA in the rat brain or neuronal cell cultures, using different molecular markers of oxidative stress, such as reduction in the levels of lipid peroxides and protein carbonyls, recycling endogenous antioxidants such as vitamin C and E, increasing glutathione levels (Packer et al., 1997; Di Domenico et al., 2015; Mehrotra et al., 2015), inhibiting free radical formation, chelating transition metal ions such as iron, thus reducing its bioaccumulation in the brain (Moini et al., 2002; Shay et al., 2009; Rochette et al., 2013). LA was also shown to display anti-inflammatory properties (Deuther-Conrad et al., 2001; Li et al., 2015), and affect cell death. *In vivo* and *in vitro* studies showed that LA ameliorated neurodegeneration in the hippocampus, decreasing neuronal apoptosis and caspase-3 protein levels, supporting a neuroprotective role mediated by the mitochondrial cell death pathway. These effects suggest that LA is able to improve mitochondrial dysfunctions. Interestingly, in addition to decreasing neuronal cell death, LA also inhibited fAβ formation from amyloid β-protein, ameliorating the neurological damage induced by Aβ, and significantly restored AChE activity. This evidence suggests that LA presents a potential role in enhancing cholinergic and cognitive functions. These neuroprotective effects may be related to the properties of LA in ameliorating memory loss associated to neurodegenerative diseases.

Remarkably, LA was able to reverse age-associated glutamatergic NMDA receptor deficits (Stoll et al., 1993), which might be centrally related to LA memory-improving effects. LA was also shown to improve the function of neurotransmitter systems, including dopamine, serotonin, and norepinephrine. Taken together, these findings provide evidence that LA can reverse loss of neurotransmitters, their receptors and responsiveness to neurotransmitters, which can underlie its effects on cognitive functions.

In summary, this review has described and discussed relevant studies investigating the effects of LA on cognition as well as its cellular and molecular effects, aiming to improve the understanding of the therapeutic potential of LA in memory loss during aging and patients suffering from neurodegenerative disorders. Although the mechanisms of action of LA are not fully understood, multiple pathways are likely to be involved in its neuroprotective properties. The memory-improving effects and neuroprotective actions of LA support its use as an adjuvant treatment for neurodegenerative disorders.

## Author contributions

PM has performed literature search and has written the first draft of the manuscript. NS has extensively revised and contributed in writing the final version of the manuscript.

### Conflict of interest statement

The authors declare that the research was conducted in the absence of any commercial or financial relationships that could be construed as a potential conflict of interest.
